# Identification of a small-molecule RPL11 mimetic that inhibits tumor growth by targeting MDM2-p53 pathway

**DOI:** 10.1186/s10020-022-00537-x

**Published:** 2022-09-07

**Authors:** Bingwu Wang, Jian Gao, Zhongjun Zhao, Xuefei Zhong, Hao Cui, Hui Hou, Yanping Zhang, Junnian Zheng, Jiehui Di, Yong Liu

**Affiliations:** 1 Cancer Institute, Xuzhou Medical University, Xuzhou, Jiangsu China; 2 Center of Clinical Oncology, Affiliated Hospital of Xuzhou Medical University, Xuzhou, Jiangsu China; 3 Jiangsu Center for the Collaboration and Innovation of Cancer Biotherapy, Cancer Institute, Xuzhou Medical University, Xuzhou, China; 4 Department of Oncology, The Second Affiliated Hospital of Xuzhou Medical University, Xuzhou, 221000 China; 5 Jiangsu Key Laboratory of New Drug Research and Clinical Pharmacy, Xuzhou Medical University, Xuzhou, 221000 China; 6 Department of Radiation and Lineberger Comprehensive Cancer Center, The University of North Carolina at Chapel Hill, 450 West Drive, Chapel Hill, USA; 7 The State Key Laboratory of Medicinal Chemical Biology, Nankai University, Tianjin, China

**Keywords:** S9, RPL11-MDM2-p53 pathway, Cell proliferation, Cell cycle arrest, Apoptosis

## Abstract

**Background:**

Targeting ribosome biogenesis to activate p53 has recently emerged as a therapeutic strategy in human cancer. Among various ribosomal proteins, RPL11 centralizes the nucleolar stress-sensing pathway by binding MDM2, leading to MDM2 inactivation and p53 activation. Therefore, the identification of MDM2-binding RPL11-mimetics would be valuable for anti-cancer therapeutics.

**Methods:**

Based on the crystal structure of the interface between RPL11 and MDM2, we have identified 15 potential allosteric modulators of MDM2 through the virtual screening.

**Results:**

One of these compounds, named S9, directly binds MDM2 and competitively inhibits the interaction between RPL11 and MDM2, leading to p53 stabilization and activation. Moreover, S9 inhibits cancer cell proliferation in vitro and in vivo. Mechanistic study reveals that MDM2 is required for S9-induced G2 cell cycle arrest and apoptosis, whereas p53 contributes to S9-induced apoptosis.

**Conclusions:**

Putting together, S9 may serve as a lead compound for the development of an anticancer drug that specifically targets RPL11-MDM2-p53 pathway.

**Supplementary Information:**

The online version contains supplementary material available at 10.1186/s10020-022-00537-x.

## Introduction

Ribosomes are extremely abundant molecular machines responsible for protein synthesis in every living cell. Ribosome biogenesis is an energy-consuming process that is tightly regulated to match cellular needs (Piazzi et al. 2019; Warner 1999). Perturbations in ribosome biogenesis can initiate ribosomal stress, and may cause ribosomopathies (Mills and Green 2017). By contrast, hyperactivated ribosome biogenesis sustains unrestricted cell growth and drives tumorigenesis. Therefore, ribosome biogenesis has great potential to function as a checkpoint for cancer cell proliferation and has recently emerged as an promising therapeutic target in cancer (Bursac et al. 2021; Pelletier et al. 2018).

As the ‘guardian of the genome’, p53 plays a pivotal role in cellular stress response networks including surveilling ribosome biogenesis (Friedel and Loewer 2022; Liu et al. 2016). p53 is typically maintained at very low levels in unstressed cells, as is achieved in great part by the E3 ligase, murine double minute 2 (MDM2) (Michael and Oren 2003; Pant et al. 2013). Ribosomal proteins (RPs) such as RPL11, RPL23, and RPL5 can bind MDM2 and activate p53 (Bursac et al. 2021; Liu et al. 2016; Zhang and Lu 2009). Till now, as many as sixteen ribosomal proteins have been identified to be involved in RP-MDM2-p53 pathway. Among them, RPL11 and RPL5 are indispensable for cells to sense ribosomal stress and activate p53 (Liu et al. 2016). Additionally, our recent work revealed that RPL11 binds MDM2 and inhibits MDM2-mediated p53 proteasomal degradation upon ribosomal stress, thereby inducing p53-driven target gene activation, leading to metabolic adaptation, cell cycle arrest, or apoptosis (Liu et al. 2014, 2016; Miliani de Marval and Zhang 2011). Of note, disruption of the RPL11-MDM2-p53 pathway remarkably accelerated oncogenic Myc-induced tumorigenesis (Macias et al. 2010), further implying that RPL11-MDM2 interaction could be an anti-cancer drug target. While it remains an attractive target for the development of anti-cancer drugs, no drug specifically targets RP-MDM2-p53 pathway so far.

One of the obstacles to impede RP-MDM2-targeting drug development is that protein–protein interactions (PPIs) are more difficult to be specifically targeted, compared to enzymes such as RNA Polymerase I (Pol I). Until recently, Zheng et al. revealed the crystal structure of the interface between human MDM2 and RPL11 (Zheng et al. 2015). In this study, we identified compound S9 as a potential inhibitor of RPL11-MDM2 interaction via molecular docking-based virtual screening. S9 directly binds MDM2 and activates p53, resulting in cell cycle arrest and apoptosis. Our findings are the first to report a small molecule that specifically targets RPL11-MDM2-p53 signaling pathway.

## Material and methods

### Molecular docking based virtual screening

Crystal structure of human MDM2 complexed with RPL11 (PDB ID: 4XXB) (Zheng et al. 2015) for molecular docking based virtual screening was obtained from the RCSB Protein Data Bank (PDB) (Berman et al. 2000). Surflex docking module in SYBYL-X2.1 (SYBYL-X2.1 is available from Tripos Associates Inc., S Hanley Rd., St. Louis, MO 631444, USA) was used for the virtual screening. The small molecule database used as the screening library was the ChemDiv database (commercially available) from TopScience Co. (Shanghai, China), which comprises more than 1 million compounds. Considering that MDM2 extensively interacted with RPL11 through an acidic domain and two zinc fingers, the region Glu293-Lys334 of MDM2 was defined as an active site for small molecule inhibitor binding. In the molecular docking calculations, the hydrogen atoms of receptor MDM2 were added and all water molecules were removed. The binding affinities of inhibitors were evaluated by docking score, the latter of which is a modified empirical scoring function based on Hammerhead. To accelerate the virtual screening, a high-speed screening was firstly carried out by decreasing the maximum quantity of conformations and rotatable bonds from 20 to 10, and from 100 to 50, respectively. Then, the molecules with docking scores within the top 1% were screened again using the default docking parameters. After two rounds of virtual screening, compounds were selected by docking score and clustering analysis.

### Antibodies and reagents

Anti-p53, anti-p21 and anti-Cdc25c mouse monoclonal antibodies were from Santa Cruz Biotechnology (CA, USA). Anti-Human PARP mouse polyclonal antibody was from BD Pharmingen (CA, USA). Anti-Flag mouse monoclonal antibody was from Sigma-Aldrich (Shanghai, China). anti-β-actin mouse monoclonal antibody was from Proteintech (Wuhan, China). Protein A Magnetic Beads and Protein G Magnetic Beads were from Thermo Scientific (Shanghai, China). Dimethyl sulfoxide was from Aladdin for dissolving S9, and S9 was dissolved with DMSO to make into 100 mM storage solution for in vitro experiments. Q-VD-OPh was obtained from Selleckchem (HOU, USA). siLenFect Lipid Reagent was purchased from Bio-Rad (Shanghai, China). Effectene Transfection Reagent was purchased from QIAGEN (Shanghai, China).

### Cell lines and growth conditions

Human osteosarcoma U2OS and colon adenocarcinoma HCT116 cell lines expressing wt p53 and its p53-null isogenic derivative were kindly provided by Dr. Yanping Zhang (The University of North Carolina at Chapel Hill, North Carolina, USA). Lung alveolar epithelial A549, renal carcinoma 786-O and ACHN, hepatoma HEPG2 cell lines were purchased from Shanghai Institute of Biochemistry and Cell Biology, Chinese Academy of Sciences (Shanghai, China). All cells were cultured in Dulbecco’s modified Eagle’s medium containing 10% fetal bovine serum, 100 U/ml penicillin, and 100 µg/ml streptomycin, in a humidified 5% CO_2_ atmosphere at 37 °C.

### Cellular thermal shift assay (CETSA)

CESTA was performed as previously described (Martinez Molina et al. 2013). Briefly, the U2OS cell lysates were collected, diluted and divided into two aliquots, treated with S9 or DMSO as control. After incubation for 30 min at room temperature, the respective lysates were divided into smaller aliquots and heated individually at different temperatures (thermal cycler, Applied Biosystems). The heated lysates were centrifuged and the supernatants were analyzed by Western blot analysis.

### Cell viability assay

Cell viability was analyzed using cell counting kit-8 (CCK-8) (Beyotime, Nantong, China). Cells were seeded in a 96-well plate at a density of 1.5 × 10^4^ cells/well and treated with a 0–800 μM concentration of S9 for 24 h. Then, 100 μl serum-free culture medium and 10 μl CCK-8 solutions were added to each well, followed by incubation at 37 °C for 1.5 h. The absorbance was measured on a microplate reader at a wavelength of 450 nm.

### Cell cycle and apoptosis

Cells were seeded in six-well plates at a 50–60% density and treated with S9 or solvent for 24 h. For cell cycle, cells were stained with propidium iodide (400 μg/ml) and RNase A (20 mg/ml) at room temperature for 30 min, samples were then analyzed using a FACSCanto flow cytometer (BD Biosciences, San Jose, CA), data on cell cycle distribution were analyzed using ModFit LT 3.0 software. For apoptosis, cells were analyzed by flow cytometry using the Annexin V-FITC/PI Apoptosis Detection Kit from KeyGEN Biotechnology (Nanjing, China) according to the manufacturer’s instructions.

### Western blot analysis

Cells were seeded in six-well plates at a 50–60% density and treated with S9 or solvent for 24 h. Protein samples from cells were extracted in 0.5%NP-40 lysis buffer and then quantified using the Brad-ford (G250) assay (KeyGEN Biotech, Nanjing, China). Samples were resolved by SDS-PAGE, then transferred to NC membranes (PALL, NY, USA), and incubated overnight at 4 °C with the primary antibody described earlier in Antibodies and Reagents. After incubation with secondary antibodies (Beyotime, Nantong, China) at 37 °C for 1.5 h, the specific proteins on NC membrane were detected by ECL kit (Advansta, CA, USA) on the Bio-Rad automatic chemiluminescence imaging system (CA, USA).

### RNA extraction and RT-PCR

Total RNA from U2OS cells treated with S9 or solvent for 24 h, was extracted using RNeasy Mini Kit (Qiagen, CA, USA). 600 ng of RNA was used for cDNA synthesis using HiScript II Q Select RT SuperMix for qPCR (Vazyme, Nanjing, China) in 20 μl final volume, following the manufacturer’s instructions. SYBR-Green reagents (Vazyme, Nanjing, China) were used for the 40-cycle real-time PCR, and RT-qPCR assays were performed using an Applied Biosystems 7500 real-time PCR machine. Specific primers for MDM2, CDKN1A(p21), Puma, Bax and p53 were were used and actin was used as a reference gene.

### Immunoprecipitation (IP)

U2OS cells were treated with different concentrations of S9 for 24 h after cotransfection with plasmids of Flag-MDM2 and MYC-L11 or not. Cell lysates were prepared in 0.1% NP-40 lysis buffer), and the supernatants were transferred to a new tube. 1 mg cell extracts were precleared with 50 µl of Sepharose CL4B for 20 min, and the supernatant was incubated with corresponding antibodies with gentle shaking at 4 °C overnight, followed by the addition of 15 μl of Pierce Classic Magnetic protein A/G-beads (Thermo Scientific, CA, USA) for another 2 h. The beads were washed three times with cold lysis buffer and then resuspended in 30 μl of 1× loading buffer and boiled for 5 min, and analyzed by SDS-PAGE.

### In vivo antitumor assays

Female BALB/c (6 weeks old) nude mice were obtained from Beijing Vital River Laboratory Animal Technology (China). Animal experiments were approved by the Animal Care Committee of Xuzhou Medical University. HCT116 (1 × 10^6^) cells were suspended in 200 μl medium containing 100 μl PBS and 100 μl Matrigel and injected subcutaneously into flank sites of mice. 7 days later, mice were randomly divided into three groups consisting of four mice each. Mice bearing xenograft tumors had an administration of S9 5 mg/kg, 25 mg/kg, or PBS as negative control by tail vein injection. Mice were injected once every other day for six times. Mice weight and tumor volumes were monitored. The tumor volume was calculated by the following formula V = L × W^2^/2, where L is the longest diameter, and W is the shortest diameter. 18 days later, three groups of mice were sacrificed and their subcutaneous tumors were dissected.

### Statistical analysis

Quantitative data are expressed as mean ± SD. Statistical analysis in treatment groups was evaluated by Student’s t-tests. All experiments were carried out at least three times unless otherwise indicated. *p < 0.05, **p < 0.01, ***p < 0.001 was considered as a statistically significant difference.

## Results

### Identification of compound S9 as a potential inhibitor of RPL11-MDM2 interactions via molecular docking based virtual screening

**Fig. 1:**
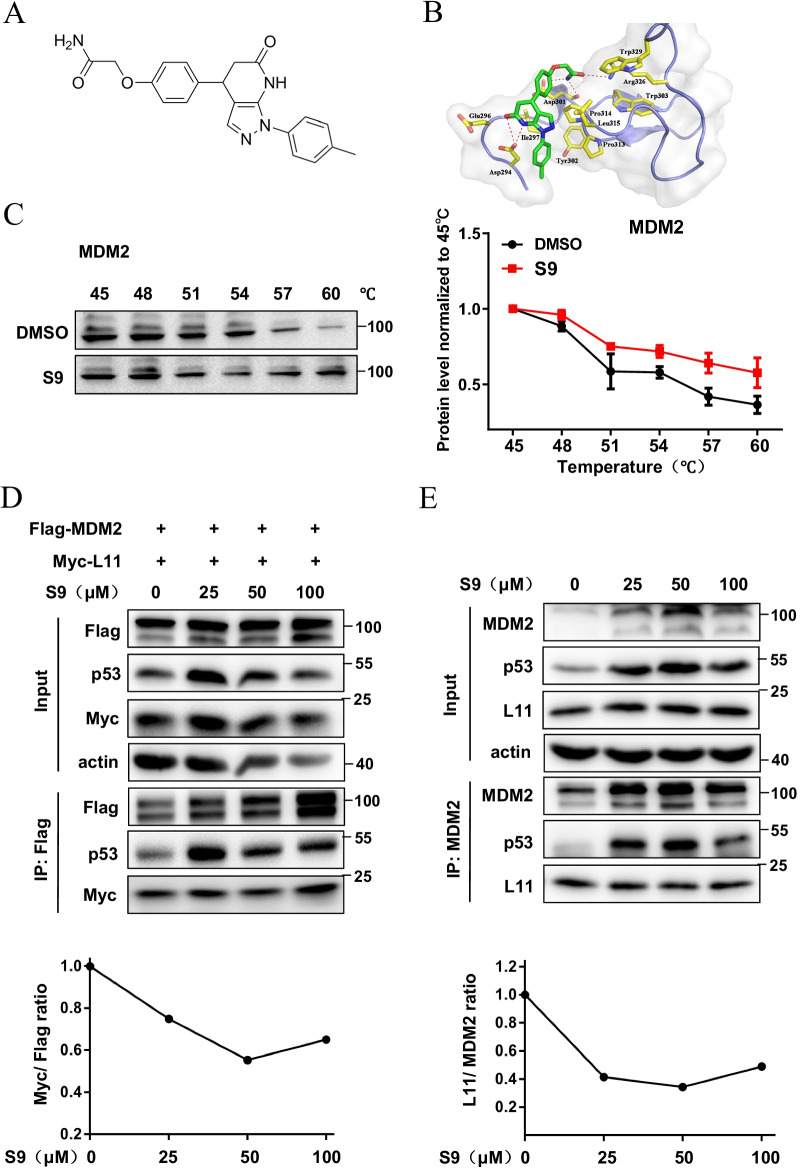
Small molecule S9 binds MDM2 and antagonizes interaction between MDM2 and RPL11. **A** Structure of compound S9 as for MDM2 inhibitor which was obtained from molecular docking based virtual screening. **B** Predicted binding mode of MDM2 complexed with compound S9. The protein MDM2 is shown in cartoon mode and colored in blue. S9 is shown in stick mode and colored in green. The key residues for S9 binding were also labeled and colored in yellow. **C** Addition of 100 μM S9 to U2OS cells followed by western blot detection of thermal stability of MDM2. The line chart beside shows the quantitation results of western blotting images. **D** U2OS cells were cotransfected with pcDNA3-Flag-MDM2 and pcDNA3-Myc-L11 followed by treatment of S9 for 24 h. Co-IP was performed with anti-Flag antibody followed by immunoblotting with anti-Flag, anti-p53 and anti-Myc antibodies. **E** U2OS cells were treated with different concentration of S9 for 24 h. Co-IP was performed with homemade anti-MDM2 antibody (2A10) followed by immunoblotting with anti-MDM2, anti-p53 and anti-L11 antibodies

After two rounds of molecular docking-based virtual screening, 15 hits were selected for the following biological activity assay. Among 15 hits, one compound named J012-3168 (S9) (Fig. 1A) stood out because of its high docking score (7.78) and potent MDM2 inhibitory activity. To depict the binding interaction between protein MDM2 and compound S9, the conformation of the MDM2/S9 complex was obtained from the molecular docking-based virtual screening (Fig. 1B). Compound S9 formed two hydrogen bonds with the side chain of residue Asp294, the latter of which is one of the key residues in the acidic domain of MDM2 for RPL11 binding via salt bridge interactions (Zheng et al. 2015). Moreover, the amide group of compound S9 also formed three hydrogen bonds with Asp301 and Arg326. These two residues were also regarded as the key residues in the binding of MDM2 with RPL11. It is likely that compound S9 had conserved hydrogen bond interactions with MDM2. In addition, compound S9 also had strong hydrophobic interactions with the residues Ile297, Tyr302, Pro313, Pro314, Leu315 and Trp329.

### S9 binds MDM2 and antagonizes interaction between MDM2 and RPL11

To validate the binding of S9 to MDM2, we performed the CETSA to determine the temperature-dependent changes of MDM2. As shown in Fig. 1C, MDM2 was stabilized by S9, confirming that MDM2 was the binding partner of S9. We also confirmed that S9 could bind mouse MDM2 in Renca cells which is a murine renal carcinoma cell line by CETSA assay (Additional file 1: Fig. S1). In addition, we performed immunoprecipitation experiments to verify whether S9 competes with L11 for binding to MDM2. Exogenous Co-IP results showed that although the immunoprecipitated Flag-MDM2 increased significantly after S9 treatment, the co-immunoprecipitated Myc-L11 level remains similar, which indicates that every unit of Flag-MDM2 binds less of Myc-L11 protein, implying that S9 has the ability to interrupt the interaction between MDM2 and L11 (Fig. 1D). Furthermore, the similar results were also observed in endogenous IP experiments (Fig. 1E). We also did Co-IP with the extra blotting of molecules in the flow-through fraction and found that L11 protein could be easily detected in flow-through, suggesting that L11 protein was not exhausted by the increased amount of MDM2 (Additional file 1: Fig. S2A). In addition, we performed immunoprecipitation experiments to verify whether Nutlin-3 can compete with L11 for binding to MDM2. Results showed that Nutlin-3 upregulate the binding efficacy between Myc-L11 and Flag-MDM2 (Additional file 1: Fig. S2B), which indicating that S9 could specifically affect RPL11-MDM2 interaction.

### S9 inhibits cancer cell growth in vitro and in vivo

**Fig. 2:**
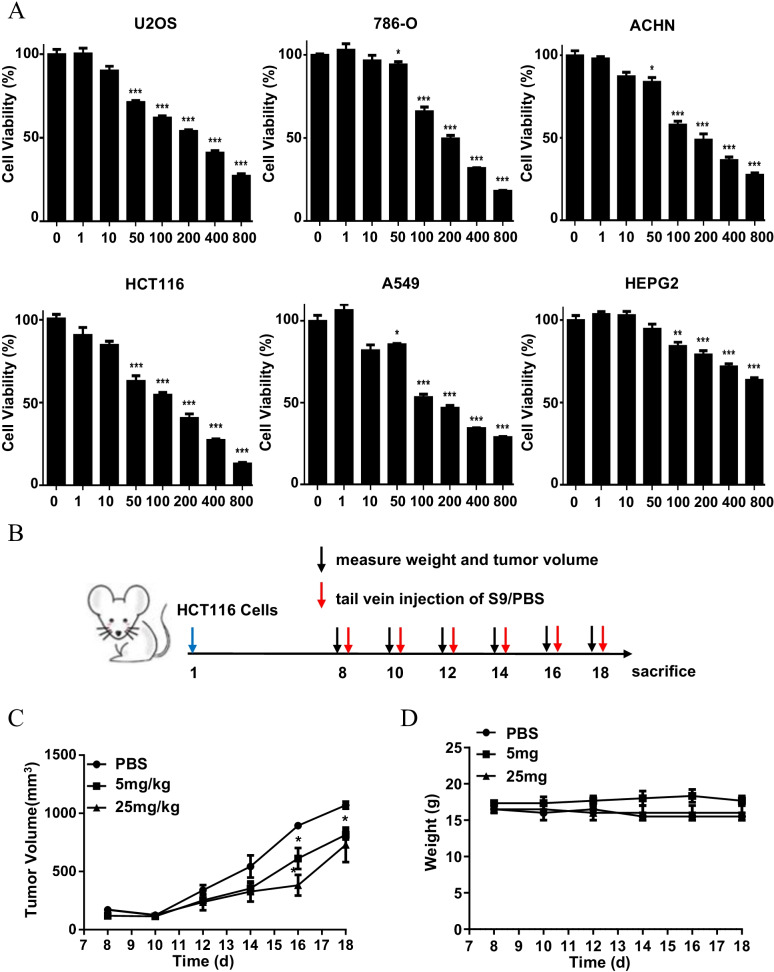
S9 inhibits cancer cell growth in vitro and in vivo. **A** U2OS, 786-O, ACHN, HCT116, A549 and HEPG2 were treated with various concentrations of S9 for 24 h. Cell Viability was measured by CCK-8 assay. The inhibition of cell proliferation was achieved by comparing the values of each group with the control. Error bars represent SDs of at least three independent measurements. **B** Treatment schedule of the in vivo antitumor experiment. **C** Relative changes in tumor volume versus time. HCT116 (5 × 10^6^) cells were injected subcutaneously into flank sites of BALB/c nude mice. Seven days later, mice were injected 5 mg/kg, 25 mg/kg S9 or PBS by tail vein injection once every other day for six times. **D** Relative changes in body weight versus time. Data represent mean ± SD (n = 4). *P < 0.05; **P < 0.01; ***P < 0.001 in comparison with PBS group

To evaluate the effects of S9 on cancer cell proliferation, we performed a CCK-8 cell proliferation assay in six different cancer cell lines including osteosarcoma U2OS, human renal carcinoma 786-O and ACHN, colon adenocarcinoma HCT116, lung alveolar epithelial A549 and hepatoma HEPG2 cell lines. As shown in Fig. 2A, a noticeable growth inhibitory effect was obtained with S9 treatment (IC 50 values of 100 μM) in five tumor cell lines. However, the growth inhibitory effect was weaker in HEPG2 cells compared with the other five cell lines. We analyzed MDM2 and RPL11 expression pattern in different cancer cells using the Cancer Dependency Map (DepMap) Portal database (Additional file 1: Fig. S3A). As shown in Fig. 2A, compared with U2OS cells, the growth inhibitory effect of S9 was weaker in HEPG2 cells which has slightly lower levels of MDM2. More importantly, we directly compared the effect of S9 in SJSA-1 cells vs U2OS cells, both of which derived from osteosacoma while SJSA-1 has a ~ 25-fold amplification of the mdm2 gene than U2OS cells (Tovar et al. 2006). As shown in Additional file 1: Fig. S3B, the growth inhibitory effect of S9 was remarkably stronger in SJSA-1 cells compared with U2OS cells. These results indicated that the growth inhibitory effect of S9 is more apparent in MDM2-overexpressed cancer cells. These results indicated that the growth inhibitory effect of S9 is more apparent in MDM2-overexpressed cancer cells. In addition, we compared the anti-proliferative effect of S9 in Fetal human colon cell line FHC and colon cancer cell line HCT116. Results showed that S9 exerted a more significant anti-proliferative effect in HCT116 than FHC (Additional file 1: Fig. S3C). Furthermore, we compared the growth inhibitory effect of S9 in two colon cancer cell lines, HT29 (with hotspot mutation of p53) and HCT116 (with wildtype p53). As shown in Additional file 1: Fig. S3D, compared with HCT116 cells, the growth inhibitory effect of S9 was weaker in HT29 cells. These results showed that S9 had significantly weaker growth inhibitory effect in cancer cells with p53 mutation versus cells with wildtype p53.

The antitumor activity of S9 in vivo was evaluated using HCT116 human xenograft models. Mice bearing xenograft tumors were injected with S9 or PBS by tail vein injection. Mice weight and tumor volumes were monitored. Results showed that the tumor volumes were significantly decreased in mice receiving S9 treatment compared with the mice receiving PBS treatment (Fig. 2B). In contrast, no significant body weight loss was observed in S9 treated mice compared to PBS control group (Fig. 2C), implying that S9 had negligible acute side effects with the amount we applied. These results suggested that S9 had the antitumor potential in the HCT116 xenograft model.

### S9 causes G2/M-phase cell cycle arrest and apoptosis in cancer cells

**Fig. 3:**
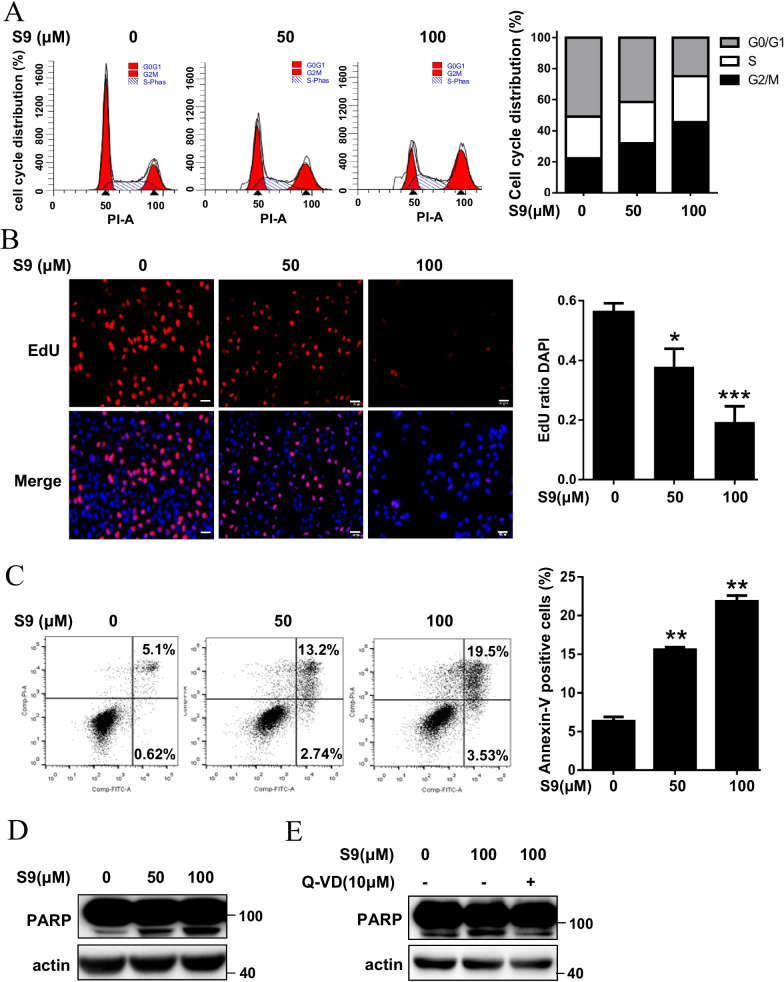
S9 causes G2/M-phase cell cycle arrest and apoptosis in cancer cells. **A** U2OS cells were treated with described concentration of S9 for 24 h, stained with PI and analyzed by flow cytometry. The dataset is representative example of triplicate experiments. Column graph was mean ± SD of three independent experiments. **B** U2OS cells were treated with described concentration of S9 for 24 h, stained with EDU and DAPI. The red color indicates EDU-positive nuclei. The statistical analysis of EDU staining was performed by Image-Pro Plus 6.0 software. The dataset is representative example of triplicate experiments. Column graph was mean ± SD of three independent experiments. **C** U2OS cells were treated with described concentration of S9 for 24 h and apoptotic cells quantitatively detected by flow cytometry. The dataset is representative example of triplicate experiments. Column graph was mean ± SD of three independent experiments. **D**, **E** U2OS cells were treated with described concentration of S9 with (**E**) or without QVD (**D**) (10 μM) for 24 h, PARP cleavage was detected by western blot analysis. The dataset is representative example of triplicate experiments. Data are presented as mean ± SD (n = 3). *P < 0.05; **P < 0.01; ***P < 0.001 in comparison with control group

To clarify whether the observed growth inhibitory effect of S9 was associated with cell cycle arrest and induction of apoptosis, we performed AnnexinV-FITC/PI assays and flow cytometry analysis to quantify the effect of S9 on cell cycle and apoptosis. As shown in Fig. 3A, S9 could induce obvious G2M-phase cell cycle arrest in U2OS cells. Similar results were also observed in HCT116 cells (Additional file 1: Fig. S4A). Furthermore, EDU staining assay showed that cell proliferation was suppressed remarkably after S9 treatment as evidenced by significantly less EdU incorporation (Fig. 3B). To clarify whether the observed growth inhibition was associated with induction of apoptosis, we performed Annexin V-FITC/PI assays and western blotting test of PARP cleavage in U2OS cells after S9 treatment. Results showed that S9 induced apoptosis in a dose-dependent manner as evidenced by the increase in Annexin V-positive cells (Fig. 3C) and PARP cleavage (Fig. 3D), and the induced apoptosis can be blocked by the pan-caspase inhibitor Q-VD-OPh (Fig. 3E). S9-induced apoptosis was also observed in HCT116 cells (Additional file 1: Fig. S4B). Therefore, these results confirmed that S9 had a noticeable growth inhibitory effect which was associated with induction of G2M-phase cell cycle arrest and apoptosis.

### S9 stabilizes p53 and increases p53 transcriptional activity

**Fig. 4:**
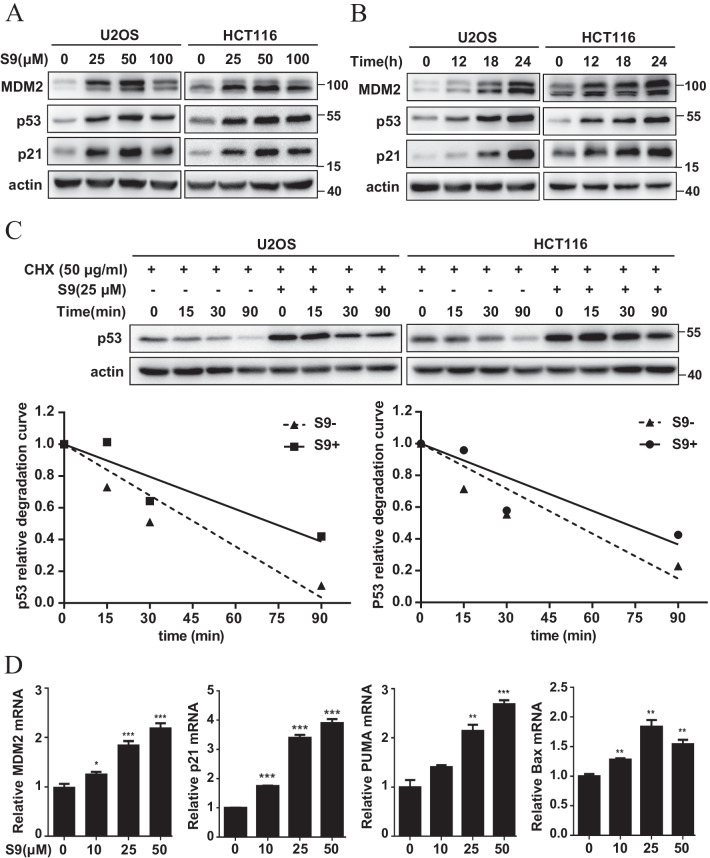
S9 stabilizes p53 and increases p53 transcriptional activity. **A** U2OS and HCT116 cells were treated with described concentration of S9 for 24 h. MDM2, p53 and p21 protein levels were detected by western blot analysis. **B** U2OS and HCT116 cells were treated with 50 μM of S9 for different time course. MDM2, p53 and p21 protein levels were detected by western blot analysis. **C** U2OS and HCT116 cells were treated with or without 25 μM of S9 for 24 h followed with cycloheximide (CHX, 50 μg/ml), and harvested at the indicated time points. p53 and actin levels were detected by western blot analysis. p53 expression normalized with actin was quantified using Image J software. **D** U2OS cells were treated with described concentration of S9 for 24 h. mRNA levels of MDM2, p21, Puma and Bax were analyzed by RT-PCR. Fold expression changes are relative to the control and correspond to mean ± SD of three independent experiments

We next evaluated the effect of S9 on the expression and activity of p53. Based on IC50 values shown previously, we treated HCT116 and U2OS cells with different concentrations and different time courses of S9. Western blotting analysis showed that S9 increased levels of MDM2, p53, and p21 in a dose-dependent and time-dependent manner (Fig. 4A, B). To determine the role of p53 on S9-induced upregulation of MDM2, we performed MDM2 immunoblotting in p53^+/+^ and p53^−/−^ U2OS cells. Results showed that S9 induced MDM2 upregulation in p53^+/+^ U2OS cells, but not in p53^−/−^ U2OS cells (Additional file 1: Fig. S5), which indicated that S9 induced upregulation of MDM2 was p53-dependent. We further examined whether S9 alters p53 protein half-life and the results presented in Fig. 4C indicate that S9 significantly increased p53 protein half-life due to p53 stabilization. It is also noted that S9 had minimal effect on p53 transcript levels (Additional file 1: Fig. S6). The activation of p53 by S9 was also demonstrated with the induction of the mRNA levels of four p53 target genes, MDM2, p21, Puma and Bax, in a dose-dependent manner (Fig. 4D). Altogether, these results demonstrate that S9 can enhance the expression, stabilization and transcriptional activity of p53.

### MDM2 is required for S9-induced p53 activation, apoptosis and cell cycle arrest

**Fig. 5:**
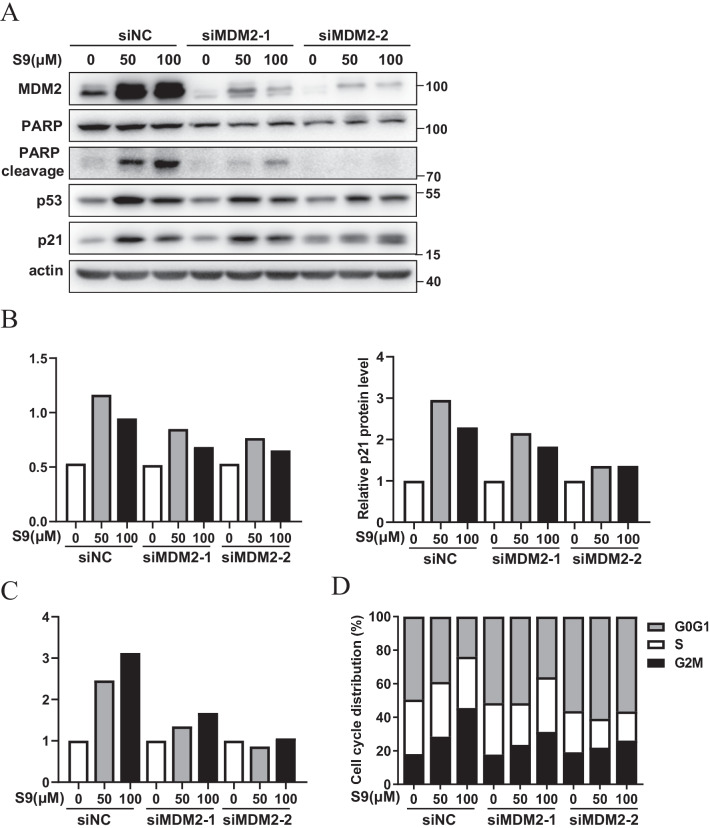
MDM2 is required for S9-induced p53 activation, apoptosis and cell cycle arrest. **A** U2OS cells were transfected with MDM2 siRNA followed with described concentration of S9 treatment for 24 h, MDM2, PARP cleavage and p53 protein levels were detected by western blot analysis. **B** The quantitation results of p53 and p21 western blotting images. **C** The quantitation results of cleaved PARP(CL)/total PARP(TL) western blotting images. **D** U2OS cells were transfected with MDM2 siRNA followed with described concentration of S9 treatment for 24 h, stained with PI and analyzed by flow cytometry

Previous experimental results showed that S9 bound to MDM2 and competed with L11 for combining with MDM2. To determine whether S9 induced cell cycle arrest and apoptosis were MDM2-dependent, we performed p21, PARP immunoblotting and cell cycle analysis in U2OS cells transfected with MDM2 siRNAs (Fig. 5A). Results showed that the increase in p53 and p21 expression (Fig. 5B), PARP cleavage (Fig. 5C) and G2M-phase cell cycle arrest (Fig. 5D) caused by S9 was abrogated, and the level of MDM2 protein was significantly reduced by two specific MDM2 siRNA (Fig. 5A). A similar result was also observed in HCT116 cells (Additional file 1: Fig. S7). These results indicated that S9 induced p53 activation, apoptosis and cell cycle arrest are MDM2 dependent.

### p53 involves in S9-induced apoptosis but not cell cycle arrest

**Fig. 6:**
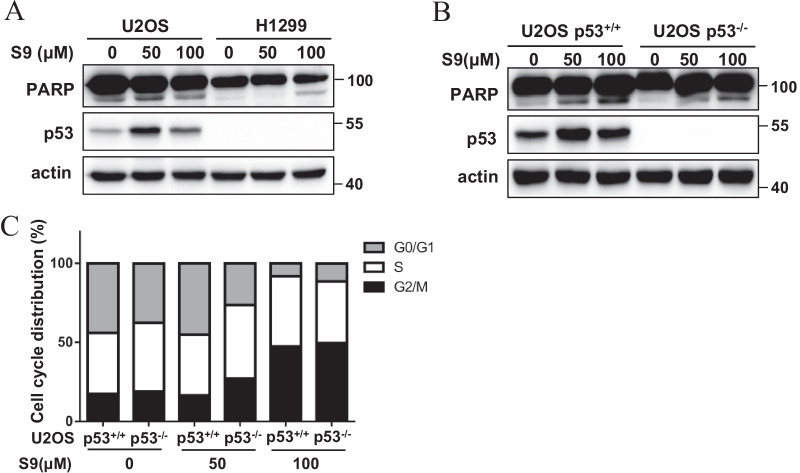
p53 involves in S9-induced apoptosis but not cell cycle arrest. **A** U2OS cells and H1299 (p53 null) cells were treated with described concentration of S9 for 24 h. PARP and p53 protein levels were detected by western blot analysis. **B**, **C** U2OS p53^+/+^ and p53^−/−^ cells were treated with described concentration of S9 for 24 h. PARP and p53 protein levels were detected by western blot analysis (**B**); stained with PI and analyzed by flow cytometry (**C**)

To determine the role of p53 on S9-induced cell cycle arrest and apoptosis, we performed PARP immunoblotting and cell cycle analysis in p53^+/+^ and p53^−/−^ U2OS cells or H1299 (p53 null) cells. Results showed that S9 induced apoptosis in a dose-dependent manner as evidenced by the increase of PARP cleavage in p53^+/+^ U2OS cells, but not in p53^−/−^ U2OS cells or H1299 cells (Fig. 6A, B). However, G2M-phase cell cycle arrest caused by S9 was not changed in U2OS p53^−/−^ cells compared with U2OS p53^+/+^ cells (Fig. 6C), which indicated that S9 induced G2M-phase cell cycle arrest was p53 independent. A similar result was also observed in HCT116 p53^+/+^ and p53^−/−^ cells (Additional file 1: Fig. S8).

## Discussion

In this study, we reported the identification of compound S9, an RPL11-mimicking p53 agonist. S9 directly binds MDM2 and antagonizes the interaction between MDM2 and RPL11, thus stabilizing p53 and increasing p53 transcriptional activity, and inducing G2/M cell cycle arrest and apoptosis. This putting together leads to significant inhibition of cancer cell growth of S9 in vitro and in vivo, which is required for MDM2 or p53. Our findings are the first to develop small molecules that specifically target and activate RP-MDM2-p53 pathway. The current results suggest that compound S9 is a potential novel therapeutic target for cancer.

As the “guardian of the genome”, p53 is frequently mutated in human cancers (Hollstein et al. 1996). Although almost half of the human cancers retain wild-type p53, the p53 pathway is often inactivated or inhibited by its main negative regulator MDM2 (Wade et al. 2013; Yin et al. 2002). The MDM2 is also the target of p53, resulting in a autoregulatory MDM2-p53 feedback loop (Haupt et al. 1997; Toledo and Wahl 2006; Yin et al. 2002). MDM2 can decrease p53 protein levels and attenuate p53 function mainly through three different mechanisms. First, MDM2 binds the transactivation domain of p53 to block its ability of transcription. Second, it promotes the export of p53 from the nucleus to the cytoplasm. Third, MDM2 acts as an E3 ubiquitin ligase mediating p53 ubiquitination and degradation, to maintain normal physiological low levels of p53 (Michael and Oren 2003). Extensive research demonstrated that inhibition of MDM2-p53 interaction to reactivate p53 has attracted significant attention in cancer therapy (Fang et al. 2020; Rusiecki et al. 2019). A number of MDM2-p53 interaction inhibitors have been identified, such as nutlin-3a (Reich et al. 1983; Wang et al. 2001) RG7112 (Tovar et al. 2013), SAH-P53-8 (Bernal et al. 2007) and ATSP-7041 (Chang et al. 2013). Although some of them have entered clinical trials, there is no molecule that passed clinical trials and got market registration so far. Mdm2-p53 inhibitors still have many side effects, including dose being limited by myelosuppression especially thrombocytopenia, metabolic disturbances, and gastrointestinal toxicities (Iancu-Rubin et al. 2014; Konopleva et al. 2020; Mahfoudhi et al. 2016).

RPs-MDM2 interaction is genuine in vivo p53 stress-signaling pathway activated by aberrant ribosome biogenesis (Macias et al. 2010). Perturbation of ribosome biogenesis to activate p53 will be one encouraging anticancer strategy. RNA Polymerase I (Pol I) is a more popular drug target to develop ribosome biogenesis-targeting therapeutic approaches, and several attempts such as CX-5461 (Bywater et al. 2012), CX-3543 (Drygin et al. 2009), and BMH-21 (Peltonen et al. 2014) have entered clinical trials (Catez et al. 2019). However, RNA Pol I is responsible for the transcription of ribosomal RNA (rRNA) which is essential for almost all life events. On the other hand, deregulation of Pol I is associated with ribosomopathies, atrophy, cardiac failure, and cancer (Hannan et al. 2013). Additionally, these tested Pol I inhibitors only have a limited selectivity towards rRNA genes, they can bind elsewhere in the genome although they preferentially affect Pol I, and the observed IC50 values of these inhibitors vary much among different cell lines (Drygin et al. 2011; Peltonen et al. 2014). Therefore, Pol I targeting strategy remains skeptical due to the comprehensive cytotoxicity and possible side-effect. In this study, we specifically focus on the downstream nucleolar stress-sensing machinery RP-MDM2-p53 pathway.

Most RPs bind to the central zinc-finger region of MDM2 to disrupt interaction with p53. Among the many MDM2-binding RPs, RPL5 and RPL11 function as both sensor and effector of ribosomal stress, while other RPs only bear the effector function and interact with RPL5 or RPL11 to regulate MDM2-p53 pathway by forming the RPs-RPL5/RPL11 complex (Kim et al. 2014; Wang et al. 2015; Zhou et al. 2012). The key positioning of RPL5 and RPL11 may also make them more sensitive to disruption in subunit assembly (Liu et al. 2016). Of note, depletion of RPL5 or RPL11, but not of other RPs, results in abrogation of ribosomal stress-induced p53 activation and cell cycle arrest (Fumagalli et al. 2012). Additionally, RPL5 and RPL11 have increased stability after ribosomal biogenesis stress compared to other RPs (Bursac et al. 2012). These findings imply that RPL5 and RPL11 play a pivotal role in p53 regulation and ribosomal stress sensing, and RPL5/RPL11-MDM2 interaction could be an attractive anti-cancer drug target. However, there is no drug specifically targeting RP-MDM2-p53 pathway so far.

One important reason is that, targeting protein–protein interactions are more difficult compared targeting enzymes such as Pol I. Until recently, Zheng et.al revealed the crystal structure of the interface between MDM2 and RPL11 (Zheng et al. 2015). This finding provides a structural basis for the development of anti-cancer drugs by targeting the RPL11 binding domain on MDM2.

In this study, we seek to find a small molecule RPL11 mimetics by using molecular docking-based virtual screening to identify compounds that can inhibit RPL11-MDM2 interaction. One compound S9 stood out because of its high docking score and potent MDM2 inhibitory activity. The conformation of the MDM2/S9 complex from the molecular docking-based virtual screening and the CESTA assay results showed that S9 binds MDM2, and the immunoprecipitation results further indicated that S9 can compete with L11 for binding MDM2. Although the co-immunoprecipitated Myc-L11 or L11 level remains similar, but the immunoprecipitated Flag-MDM2 or MDM2 increased significantly after S9 treatment, which indicates that every unit of Flag-MDM2/MDM2 binds less of Myc-L11 or L11 protein. In addition, we confirmed whether the similar level of co-precipitated L11 in cells treated with S9 was because of the saturated MDM2-L11 interaction. Firstly, the results of Co-IP with the extra blotting of molecules in the flow-through fraction indicating that L11 protein was not exhausted by the increased amount of MDM2 (Additional file 1: Fig. S2A). Secondly, while we treated U2OS cells with Nutlin-3, we found that Nutlin-3 could effectively enhance the level of precipitated Myc-L11 (Additional file 1: Fig. S2B). This result implies that the Flag-MDM2/Myc-L11 interaction was not saturated in control samples with no Nutlin-3 treatment, and instead, it could be augmented by Nutlin-3 treatment. Given that the two experiments in Fig. 1D and Additional file 1: Fig. S2B shared identical untreated control, our experiments suggest that MDM2-L11 interaction was not saturated in the tested system. Furthermore, we demonstrated that S9 exhibits an anticancer growth effect both in vitro and in vivo.

To further explore the molecular mechanism of anticancer activity of S9, we found that MDM2 is required for S9-induced p53 activation, apoptosis and cell cycle arrest. However, p53 involves in S9-induced apoptosis but not cell cycle arrest. Giono et al. reported that MDM2 interacts with Cdc25C which is an important player at the G2/M transition (Donzelli and Draetta 2003) and MDM2 promotes proteasome-mediated degradation of Cdc25C in a p53-independent manner (Giono et al. 2017; Piwnica-Worms 1999), suggesting that S9 could possibly downregulate Cdc25C by upregulating MDM2 and further induce G2M-phase cell cycle arrest. Indeed, we tested the Cdc25c level in U2OS after S9 treatment and found that S9 can downregulate Cdc25c both in p53^+/+^ and p53^−/−^ cells (Additional file 1: Fig. S9), whether S9 induces G2M-phase cell cycle arrest is Cdc25c dependent or not will be further explored in our future work. This might be the reason why p53-mutant cancer cells are less sensitive than cells with WT p53. In addition, our results indicated that the growth inhibitory effect of S9 is more apparent in MDM2-overexpressed cancer cells (Additional file 1: Fig. S4).

In addition, the water solubility of S9 is very low even though we tried applying cyclodextrin conjugation to increase its solubility. This made it difficult to be applied in vivo with an optimal concentration, indicating that S9 may serve as a drug lead for future modification and for the development of a safe and effective p53-targeting anti-cancer drug.

## Conclusions

In conclusion, we reported a small-molecule RPL11 mimetic S9, that directly binds MDM2 and activates p53, resulting in cell cycle arrest and apoptosis. Putting together, S9 may serve as a lead compound for the development of an anticancer drug that specifically targets RPL11-MDM2-p53 pathway.

## Supplementary Information

**Additional file 1: Figure S1.** S9 binds MDM2 in Renca cells. Addition of 100 μM S9 to Renca cells followed by western blot detection of thermal stability of MDM2. **Figure S2.** S9 antagonizes interaction between MDM2 and RPL11. (A) U2OS cells were treated with indicated concentration of S9 for 24 h. Co-IP was performed with homemade anti-MDM2 antibody (2A10) followed by immunoblotting with anti-MDM2, anti-p53 and anti-L11 antibodies. (B) U2OS cells were cotransfected with pcDNA3-Flag-MDM2 and pcDNA3-Myc-L11 followed by treatment of Nutlin-3 for 24 h. Co-IP was performed with anti-Flag antibody followed by immunoblotting with anti-Flag and anti-Myc antibodies. **Figure S3.** The anti-proliferative effect of S9 in different cancer cells. (A) MDM2 and RPL11 expression pattern were analyzed in different cancer cells using the DepMap Portal database. (B–D) SJSA-1 and U2OS cells (B), FHC and HCT116 cells (C), HT29 and HCT116 cells (D) were treated with various concentrations of S9 for 24 h. Cell Viability was measured by CCK-8 assay. The inhibition of cell proliferation was achieved by comparing the values of each group with the control. Error bars represent SDs of at least three independent measurements. **Figure S4.** S9 induces cell cycle arrest and apoptosis in HCT116 cells. (A) HCT116 cells were treated with described concentration of S9 for 24 h, stained with PI and analyzed by flow cytometry. The dataset is representative example of triplicate experiments. Column graph was mean ± SD of three independent experiments. (B) HCT116 cells were treated with described concentration of S9 for 24 h, PARP cleavage was detected by western blot analysis. **Figure S5.** S9 induced upregulation of MDM2 was p53 dependent. U2OS p53^+/+^ and p53^−/−^ cells were treated with described concentration of S9 for 24 h, MDM2 and p53 protein levels were detected by western blot analysis. **Figure S6.** S9 has minimal effect on p53 transcript level. U2OS cells were treated with described concentration of S9 for 24 h, and the mRNA level of p53 was analyzed by RT-PCR. Fold expression changes are relative to the control and correspond to mean ± SD of three independent experiments. **Figure S7.** MDM2 is required for S9-induced cell cycle arrest in HCT116 cells. (A) HCT116 cells were transfected with MDM2 siRNA, 48 h after transfection, MDM2 protein levels was detected by western blot analysis. (B) HCT116 cells were transfected with MDM2 siRNA followed with described concentration of S9 treatment for 24 h, stained with PI and analyzed by flow cytometry. **Figure S8.** p53 is required for S9-induced apoptosis but not cell cycle arrest in HCT116 cells. HCT116 p53^+/+^ and p53^−/−^ cells were treated with described concentration of S9 for 24 h, PARP and p53 protein levels were detected by western blot analysis (A); stained with PI and analyzed by flow cytometry (B). **Figure S9.** S9 downregulates Cdc25c independent of p53. U2OS p53^+/+^ and p53^−/−^ cells were treated with described concentration of S9 for 24 h, MDM2 and Cdc25c protein levels were detected by western blot analysis.

## Data Availability

The data and materials presented in this study are available on request from the corresponding author.

## Abbreviations

MDM2: Murine double minute 2
PPIs: Protein–protein interactions
PDB: Protein Data Bank
CETSA: Cellular thermal shift assay
IP: Immunoprecipitation
CCK-8: Cell counting kit-8
Pol I: RNA Polymerase I
DepMap: Cancer Dependency Map
