# Supplementary material for: Identification of a small-molecule RPL11 mimetic that inhibits tumor growth by targeting MDM2-p53 pathway
Source: Mol Med. 2022 Sep 7;28:109. doi: 10.1186/s10020-022-00537-x (PMC9450376; doi:10.1186/s10020-022-00537-x)
Supplement: Supplementary file 1 — Additional file 1: Figure S1. S9 binds MDM2 in Renca cells. Addition of 100 μM S9 to Renca cells followed by western blot detection of thermal stability of MDM2. Figure S2. S9 antagonizes interaction between MDM2 and RPL11. (A) U2OS cells were treated with indicated concentration of S9 for 24 h. Co-IP was performed with homemade anti-MDM2 antibody (2A10) followed by immunoblotting with anti-MDM2, anti-p53 and anti-L11 antibodies. (B) U2OS cells were cotransfected with pcDNA3-Flag-MDM2 and pcDNA3-Myc-L11 followed by treatment of Nutlin-3 for 24 h. Co-IP was performed with anti-Flag antibody followed by immunoblotting with anti-Flag and anti-Myc antibodies. Figure S3. The anti-proliferative effect of S9 in different cancer cells. (A) MDM2 and RPL11 expression pattern were analyzed in different cancer cells using the DepMap Portal database. (B–D) SJSA-1 and U2OS cells (B), FHC and HCT116 cells (C), HT29 and HCT116 cells (D) were treated with various concentrations of S9 for 24 h. Cell Viability was measured by CCK-8 assay. The inhibition of cell proliferation was achieved by comparing the values of each group with the control. Error bars represent SDs of at least three independent measurements. Figure S4. S9 induces cell cycle arrest and apoptosis in HCT116 cells. (A) HCT116 cells were treated with described concentration of S9 for 24 h, stained with PI and analyzed by flow cytometry. The dataset is representative example of triplicate experiments. Column graph was mean ± SD of three independent experiments. (B) HCT116 cells were treated with described concentration of S9 for 24 h, PARP cleavage was detected by western blot analysis. Figure S5. S9 induced upregulation of MDM2 was p53 dependent. U2OS p53+/+ and p53−/− cells were treated with described concentration of S9 for 24 h, MDM2 and p53 protein levels were detected by western blot analysis. Figure S6. S9 has minimal effect on p53 transcript level. U2OS cells were treated with described concentration of S9 for 24 [file 10020_2022_537_MOESM1_ESM.pptx]

## Slide 1
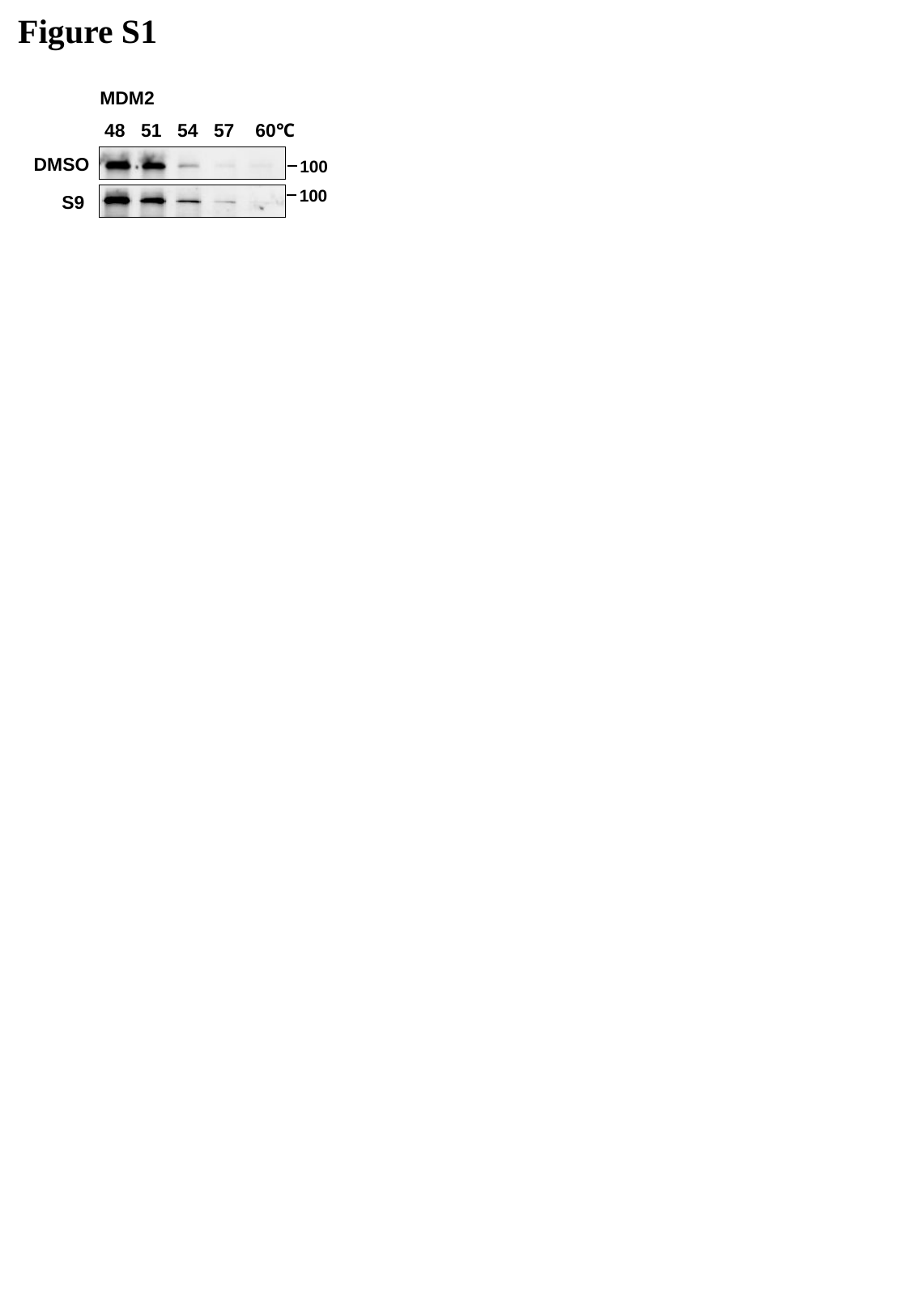

Figure S1
MDM2
DMSO
S9
48 51 54 57 60℃
100
100

## Slide 2
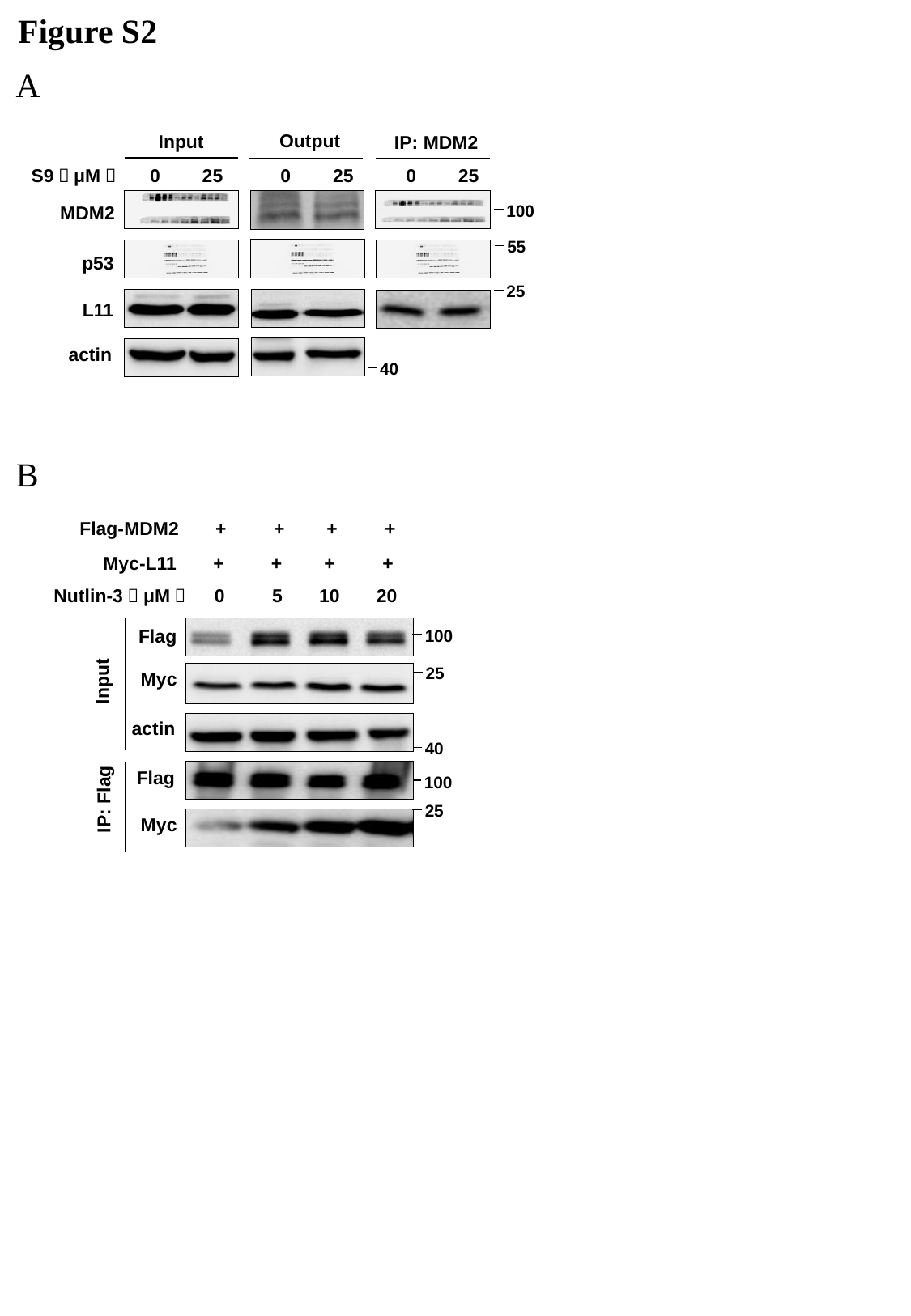

Figure S2
A
Output
Input
IP: MDM2
 S9（μM） 0 25 0 25 0 25
MDM2
p53
L11
actin
100
55
25
40
B
Flag-MDM2 + + + +
 Myc-L11 + + + +
 Nutlin-3（μM） 0 5 10 20
Flag
Myc
 Input
actin
Flag
 IP: Flag
Myc
100
25
40
100
25

## Slide 3
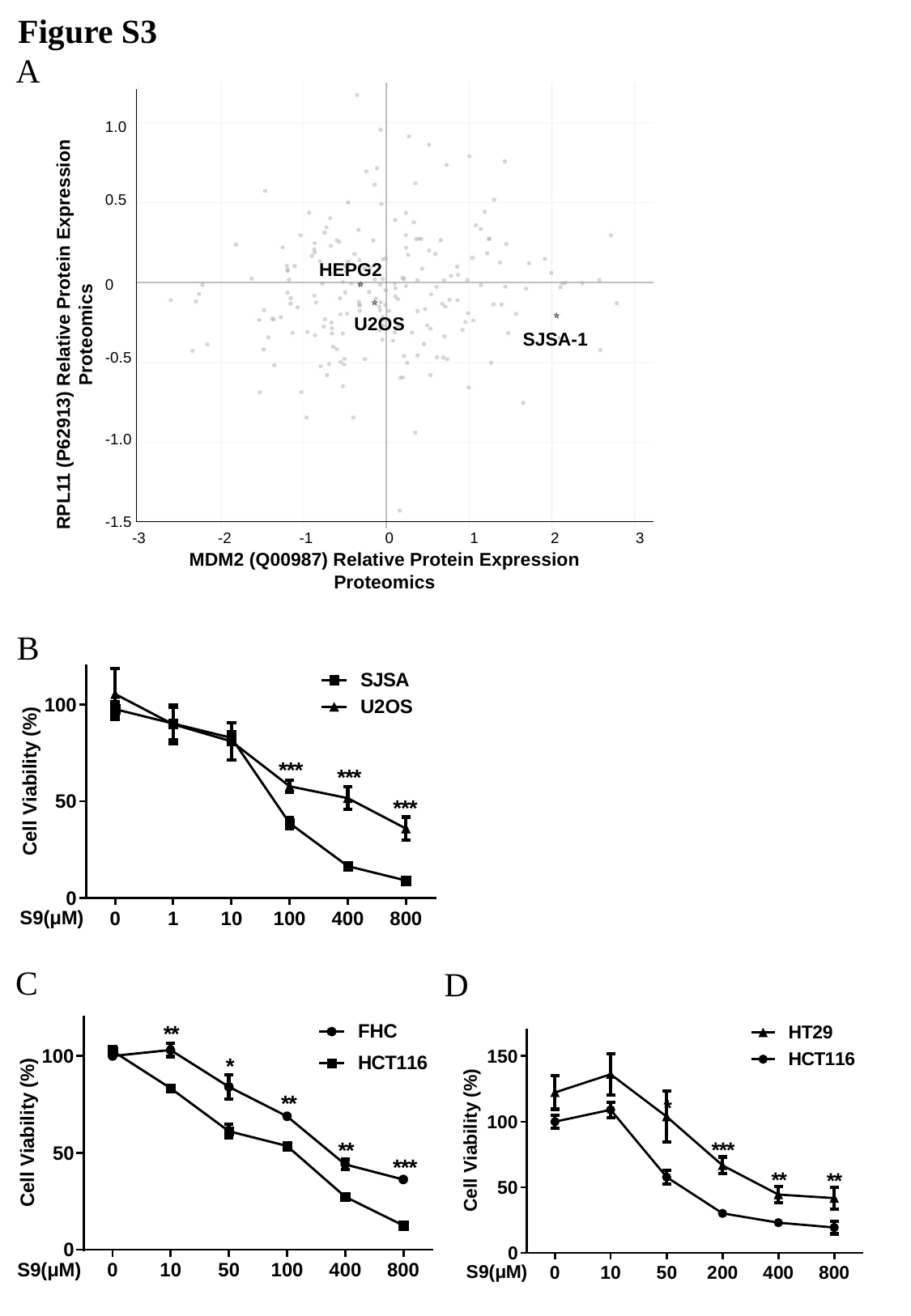

Figure S3
A
RPL11 (P62913) Relative Protein Expression
Proteomics
MDM2 (Q00987) Relative Protein Expression
Proteomics
HEPG2
U2OS
SJSA-1
1.0
0.5
0
-0.5
-1.0
-1.5
-3 -2 -1 0 1 2 3
B
C
D

## Slide 4
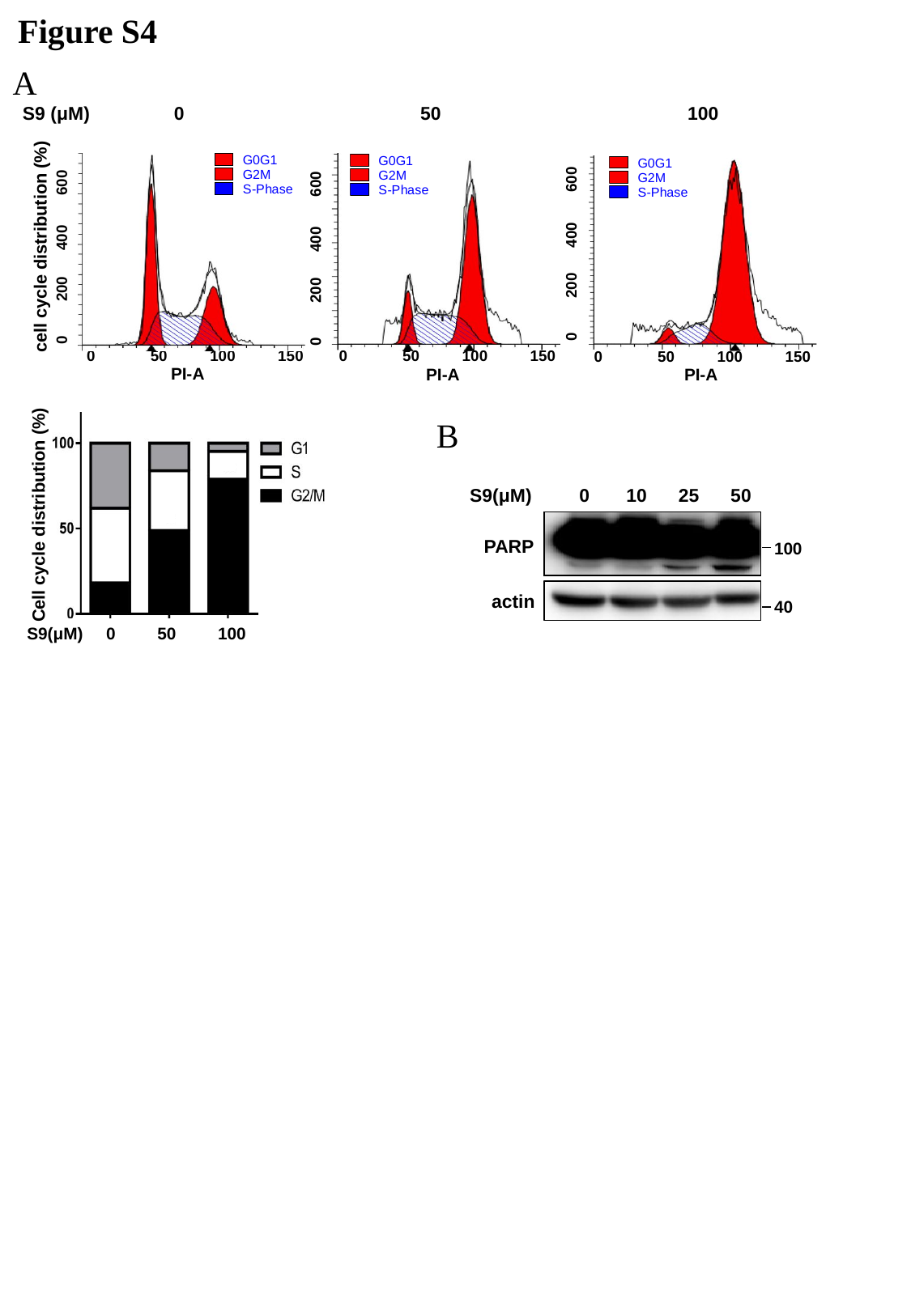

Figure S4
A
 S9 (μM) 0 50 100
 0 200 400 600
 0 50 100 150
 PI-A
cell cycle distribution (%)
 0 200 400 600
 0 50 100 150
 PI-A
 0 200 400 600
 0 50 100 150
 PI-A
Cell cycle distribution (%)
S9(μM) 0 50 100
B
S9(μM) 0 10 25 50
PARP
actin
100
40

## Slide 5
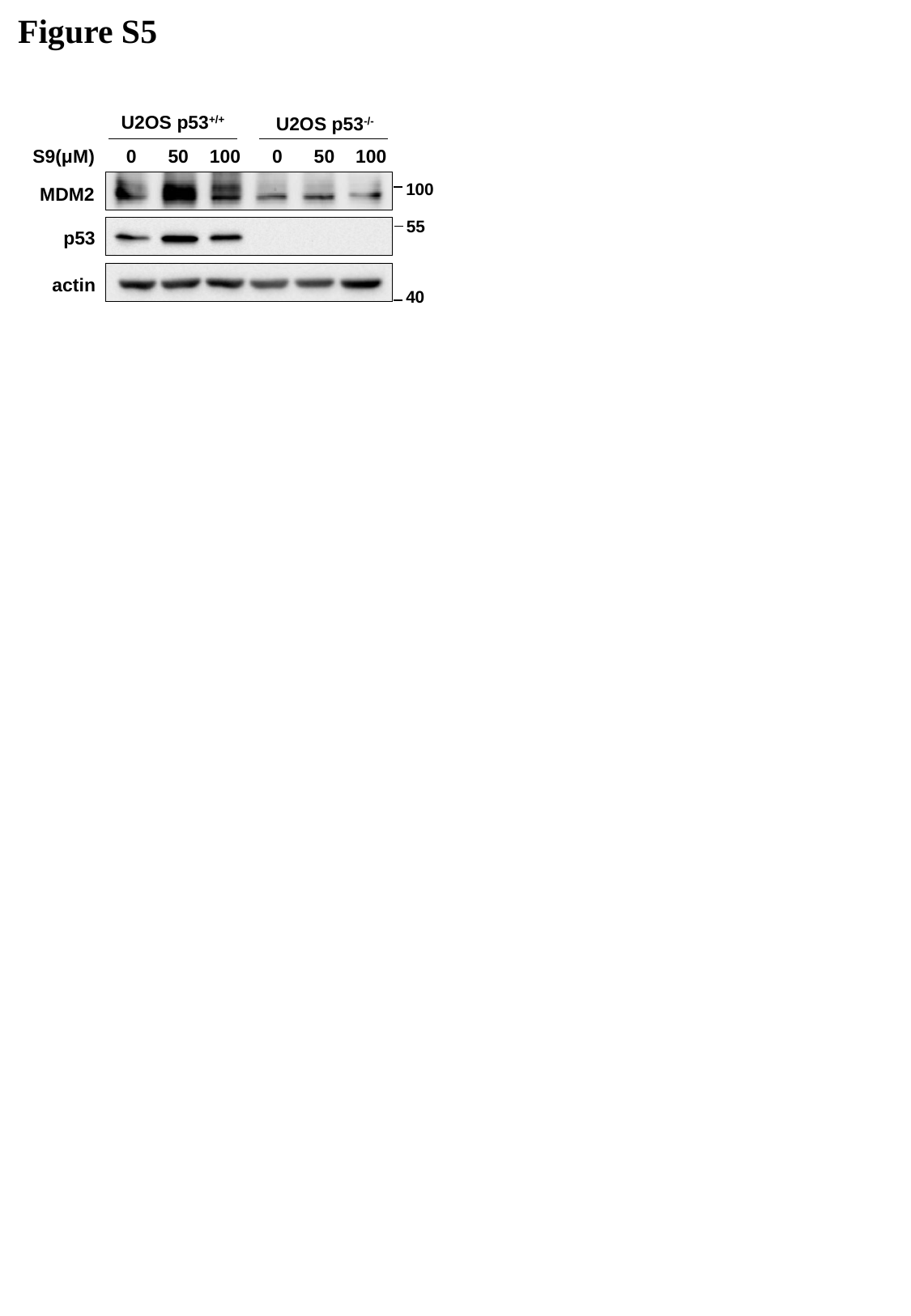

Figure S5
U2OS p53+/+
U2OS p53-/-
S9(μM) 0 50 100 0 50 100
MDM2
p53
actin
100
55
40

## Slide 6
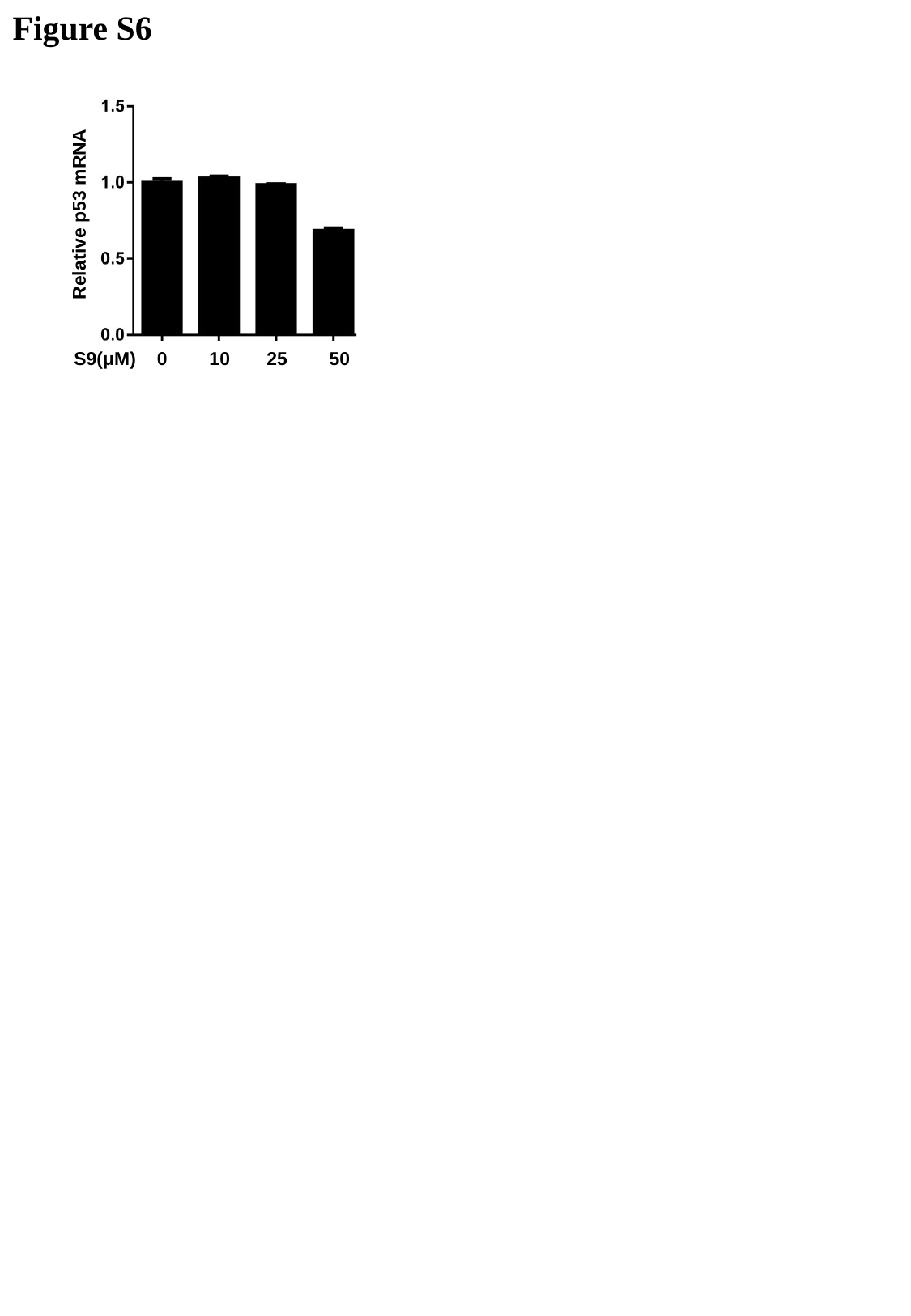

Figure S6
S9(μM) 0 10 25 50
Relative p53 mRNA

## Slide 7
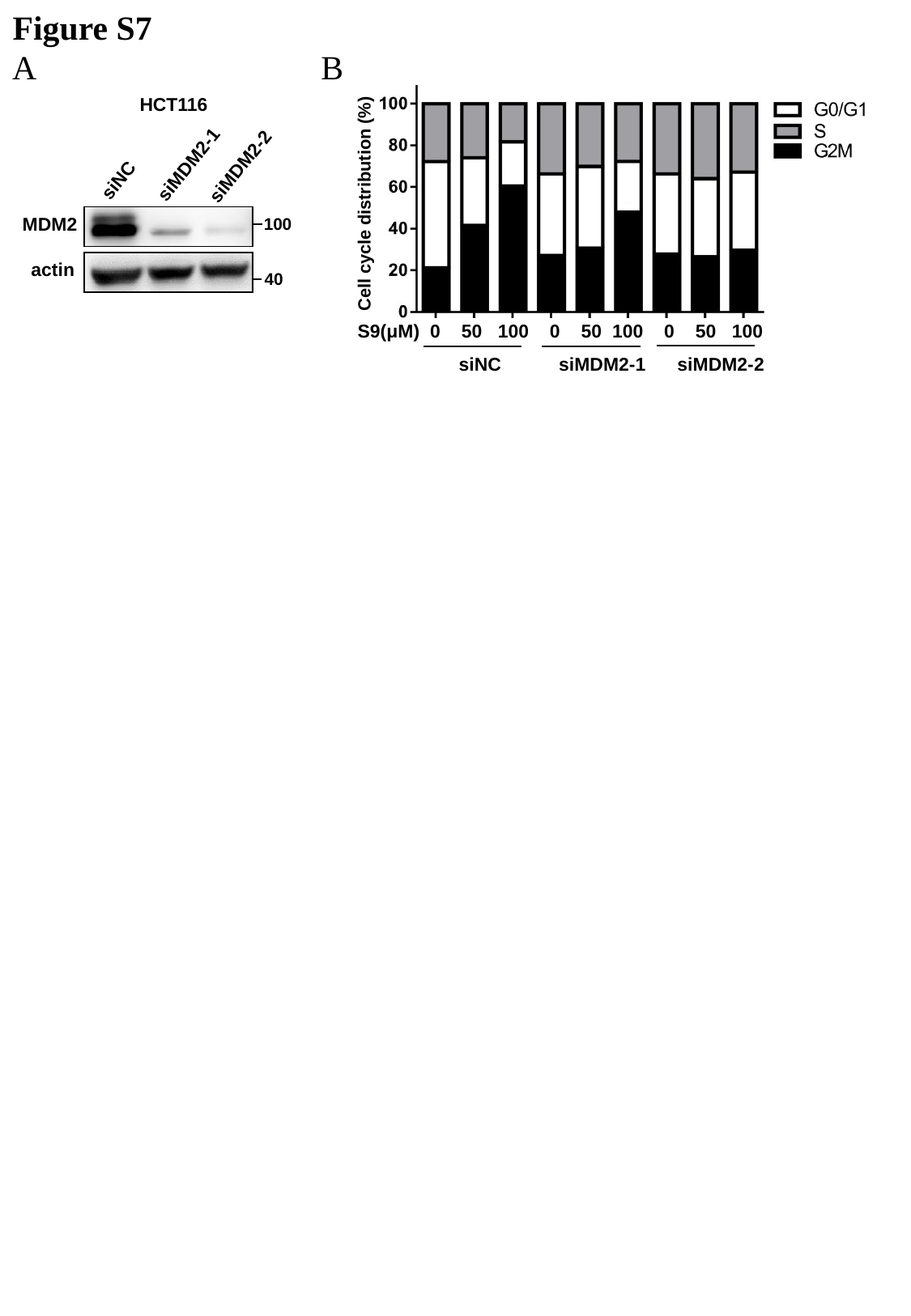

Figure S7
A
B
S9(μM) 0 50 100 0 50 100 0 50 100
 siNC siMDM2-1 siMDM2-2
 HCT116
siNC
siMDM2-1
siMDM2-2
MDM2
actin
Cell cycle distribution (%)
100
40

## Slide 8
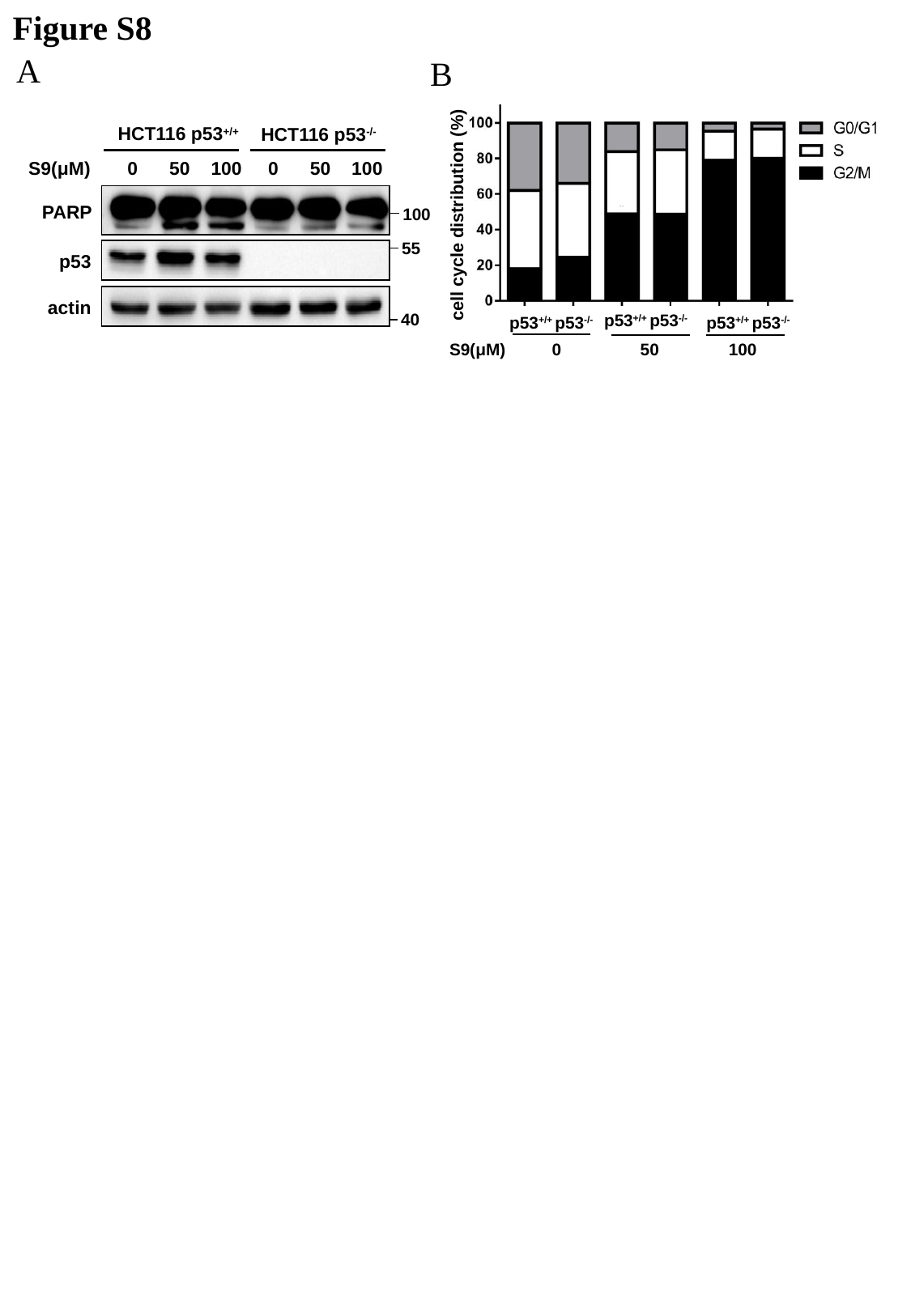

Figure S8
A
B
p53+/+ p53-/-
S9(μM) 0 50 100
p53+/+ p53-/-
p53+/+ p53-/-
cell cycle distribution (%)
 HCT116 p53+/+
 HCT116 p53-/-
S9(μM) 0 50 100 0 50 100
PARP
p53
actin
100
55
40

## Slide 9
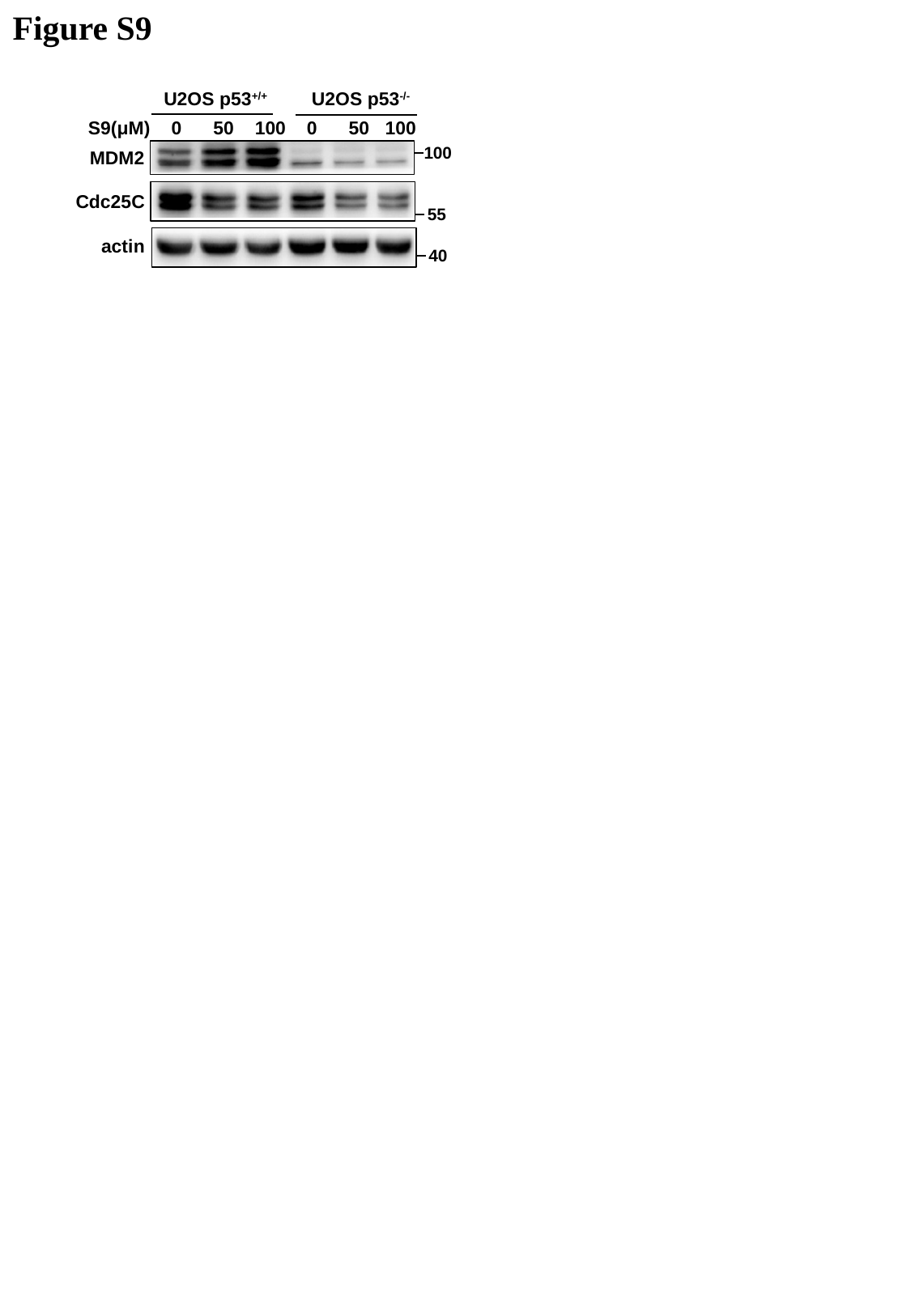

Figure S9
 U2OS p53+/+
 U2OS p53-/-
 S9(μM) 0 50 100 0 50 100
MDM2
Cdc25C
actin
100
55
40
